# Dipstick proteinuria and risk of type 2 diabetes mellitus: a nationwide population-based cohort study

**DOI:** 10.1186/s12967-021-02934-y

**Published:** 2021-06-26

**Authors:** Jimin Jeon, Jinkwon Kim

**Affiliations:** Department of Neurology, Yongin Severance Hospital, Yonsei University College of Medicine, 363, Dongbaekjukjeon-daero, Giheung-gu, Yongin, 16995 Republic of Korea

**Keywords:** Chronic Kidney Disease, Cohort, Dipstick proteinuria, Type 2 diabetes mellitus

## Abstract

**Background:**

Proteinuria has been recognized as a marker of systemic inflammation and endothelial dysfunction associated with insulin resistance and β-cell impairment, which can contribute to the development of type 2 diabetes mellitus (T2DM). However, it is unknown whether the dipstick proteinuria test has a predictive value for new-onset T2DM.

**Methods:**

This retrospective cohort study analyzed 239,287 non-diabetic participants who participated in the Korean nationwide health screening program in 2009–2010. Proteinuria was determined by the urine dipstick test at the baseline health screening. We performed multivariate Cox proportional regression analyses for the development of new-onset T2DM. Follow-up was performed until December 2015.

**Results:**

During the mean follow-up period of 5.73 years, 22,215 participants were diagnosed with new-onset T2DM. The presence of proteinuria was significantly associated with an increased risk of T2DM (adjusted hazard ratio: 1.19, 95% confidence interval: 1.10, 1.29). There was a positive dose–response relationship between the degree of dipstick proteinuria and T2DM risk. This significant association between proteinuria and T2DM risk was consistent regardless of the fasting glucose level at baseline.

**Conclusions:**

Dipstick proteinuria is a significant risk factor for new-onset T2DM. Therefore, proteinuria might be a useful biomarker to identify those at a high risk for developing T2DM.

**Supplementary Information:**

The online version contains supplementary material available at 10.1186/s12967-021-02934-y.

## Background

Type 2 diabetes mellitus (T2DM), a chronic metabolic disorder characterized by hyperglycemia with abnormal glucose regulation, is a major public health concern due to its high prevalence and increasing disease burden [1, 2]. Since T2DM is considered to be largely preventable, developing strategies to prevent T2DM and its complications is an important health topic [3]. For the prevention of T2DM, identifying risk factors and screening at-risk populations are crucial steps [4].

Proteinuria is the presence of excess proteins in the urine and has been primarily recognized as a marker of kidney damage and a predictor of future decline in renal function [5]. Increased inflammatory response and endothelial dysfunction are representative clinical features of proteinuria [6]. The systemic inflammation and endothelial dysfunction are common features of diabetes and are closely related to insulin resistance [7, 8]. Regarding inflammatory markers and endothelial dysfunction frequently precede the development of T2DM and they are predictive biomarkers for T2DM, we hypothesized that proteinuria may be an independent risk factor for new-onset T2DM [9–12]. The urine dipstick test for proteinuria is widely used in clinical practice and public health screenings because of its low cost, ease of use, rapid results, and acceptable accuracy [13, 14]. There were some prior studies for the association between proteinuria and the development of T2DM [15–17]. However, role of dipstick proteinuria on risk for T2DM is not well-known, and few studies have investigated in Korea. In the current study, we aimed to investigate whether non-diabetic participants with dipstick proteinuria are at an increased risk of new-onset T2DM using a Korean nationwide population-based cohort database.

## Methods

### Data source and study design

**Fig. 1:**
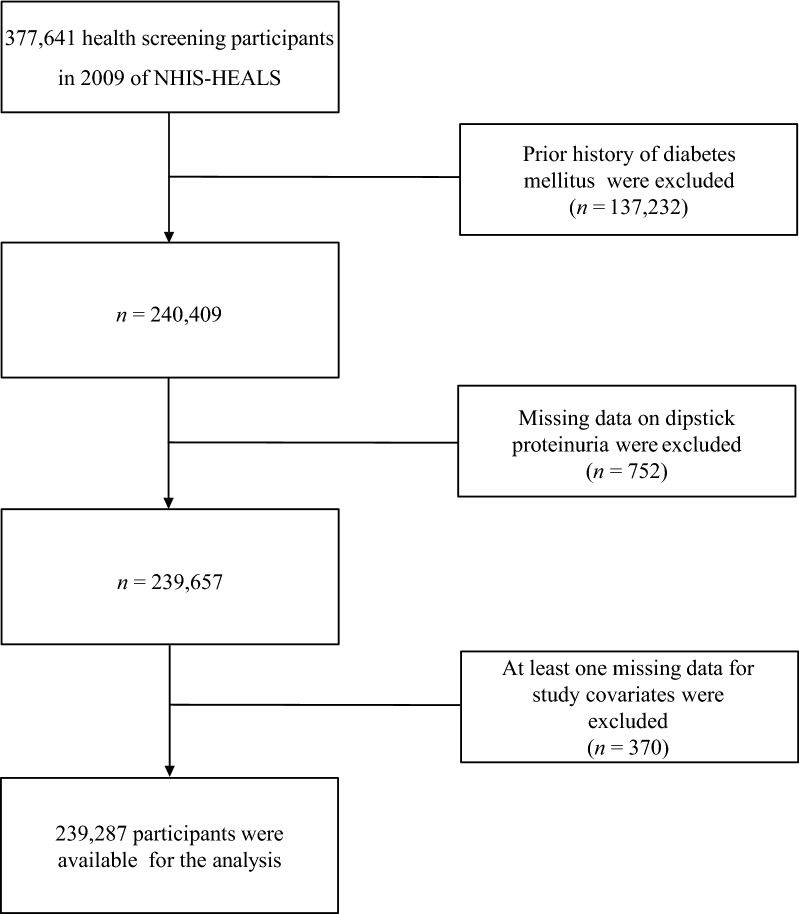
Flowchart of Included participants. NHIS-HEALS, the National Health Insurance Service-Health Screening Cohort

Current study had a retrospective cohort design and used data from the National Health Insurance Service-Health Screening Cohort (NHIS-HEALS) in Korea. Information on the dataset has been reported in detail elsewhere [18]. Briefly, the NHIS-HEALS comprises approximately 51 million Korean adults through a random selection of 10% of all health screening participants aged 40–79 years. The NHIS-HEALS contains the participants’ demographics, health claims data, death statistics, and serial health screening program results. The health screenings were conducted every 2 years between 2002 and 2015 and included a physical examination, lifestyle survey, laboratory tests including fasting serum glucose levels, and dipstick urinalysis for proteinuria. From the health claims data, the participants’ diagnoses, made at each hospital, were recorded according to the International Statistical Classification of Diseases and Related Health Problems 10th Revision. Data from the NHIS-HEALS are fully anonymized. The study participants were non-diabetics who underwent a health examination in 2009–2010 (baseline health examination). We excluded participants who had a history of diabetes and those who had no dipstick proteinuria data or covariate data from the baseline health examination. A flow chart of the inclusion process is shown in Fig. 1. From the date of baseline health examination (index date), the participants were followed up until the development of new-onset T2DM (primary outcome), death, loss of eligibility for NHIS due to emigration, or December 2015, whichever occurred earliest. This study was approved by the Institutional Review Board of Yongin Severance Hospital (9–2020-0106), and the need for informed consent was waived due to its retrospective nature and analysis with anonymized data.

### Proteinuria and study outcome assessment

As part of the health screening program, a dipstick urinalysis was performed using urine samples obtained in the morning after overnight fasting. Proteinuria was determined through an interpretation of the dipstick urinalysis results based on a color scale that quantifies proteinuria as ‘negative’, ‘trace (±)’, ‘1 + ’, ‘2 + ’, ‘3 + ’, or ‘4 + ’. For this study, we classified the dipstick proteinuria results into three groups: ‘no proteinuria (-)’, ‘trace (±)’, and ‘overt proteinuria (≥ 1 +)’. The incidence of primary outcome (new-onset T2DM) was defined as the presence of one of the following two conditions: a health claim code of E11-E14 with the prescription of any anti-diabetic drug (insulin, sulfonylurea, metformin, meglitinide, thiazolidinedione, dipeptidyl peptidase-4 inhibitor, or α-glucosidase inhibitor) during follow-up or a fasting serum glucose level ≥ 126 mg/dL obtained from the serial health screening program data [19, 20].

### Covariates

Demographic and lifestyle characteristics including sex, age, current smoking, alcohol consumption, physical activity, and family history of diabetes were collected from self-reported questionnaires at the health screening. Alcohol consumption was categorized into < 1 time, 1–2 times, 3–4 times, and ≥ 5 times based on weekly intake frequencies on average. Physical activity was divided into < 1 day, 1–4 days, and ≥ 5 days per week, according to the number of days of exercise on average. The presence of hypertension was determined if participants had a systolic/diastolic blood pressure ≥ 140/90 mmHg or answered “yes” in the corresponding health screening questionnaire concerning a diagnosis of hypertension. Body mass index was calculated by dividing the body weight (kg) by the height squared (m^2^), then categorized into underweight (< 18.5 kg/m^2^), normal (18.5–23 kg/m^2^), overweight (23–25 kg/m^2^), or obese (≥ 25 kg/m^2^) using the body mass index criteria of the World Health Organization for Asians [21]. Estimated glomerular filtration rate was calculated using the Modification of Diet in Renal Disease formula: Estimated glomerular filtration rate (mL/min/1.73 m^2^) = 186.3 × (serum creatinine^—1.154^) × (age^—0.203^) × (0.742) for women [22]. Fasting glucose levels at baseline were divided into two groups: normal fasting glucose (< 100 mg/dL) and impaired fasting glucose (≥ 100 mg/dL and < 126 mg/dL), in accordance with the American Diabetes Association criteria [23].

### Statistical analyses

Categorical variables are presented as numbers (%), and continuous variables are presented as mean (standard deviation). Differences between groups were compared using the Mantel–Haenszel test for linear trend for categorical variables and the Jonckheere’s trend test for continuous variables. We illustrated Kaplan–Meier curves for event-free survival for T2DM development according to dipstick proteinuria, which were compared using a log-rank test. Cox proportional hazard regression analysis was performed to estimate the hazard ratio (HR) and 95% confidence interval (CI) for the risk of T2DM. In the multivariate Cox models, adjustments were made for sex, age, current smoking, physical activity, alcohol consumption, family history of diabetes, body mass index, systolic blood pressure, fasting glucose level, and estimated glomerular filtration rate at baseline. The SAS version 9.4 (SAS Inc., Cary, NC, USA) and R software version 3.3.3 (The R Foundation for Statistical Computing, Vienna, Austria; http://www.R-project.org/) were used to manipulate the data and perform all statistical analyses. The statistically significant level for all tests was defined as two-sided *P* < 0.05.

## Results

### Baseline characteristics

In accordance with the inclusion and exclusion criteria (Fig. 1), 239,287 participants were included in the study. The baseline characteristics of the participants according to degree of proteinuria are shown in Table 1. Among the 239,287 participants, 95.9% (n = 229,426) did not have proteinuria, 2.1% (n = 5,054) had trace proteinuria (±), and 2.0% (n = 4,807) had overt proteinuria (≥ 1 +). The degree of proteinuria was positively associated with sex, current smoking, alcohol consumption, and obesity. A significantly increasing and decreasing trend according to the degree of proteinuria was observed for the level of systolic blood pressure, fasting glucose and estimated glomerular filtration rate at baseline, respectively.

**Table 1:** Baseline characteristics of participants according to degree of proteinuria

Variable	Total	Degree of proteinuria			*P* for trend^†^
		No proteinuria	Trace proteinuria (±)	Overt proteinuria (≥ 1 +)	
N	239,287	229,426	5054	4807	
Sex, male	128,780 (53.82)	123,210 (53.70)	2881 (57.00)	2689 (55.94)	< 0.001
Age, years	57.12 ± 8.33	57.09 ± 8.31	57.10 ± 8.68	58.50 ± 9.05	< 0.001
Current smoking	42,824 (17.90)	40,863 (17.81)	1014 (20.06)	947 (19.70)	< 0.001
Alcohol consumption, frequency per week					< 0.001
< 1 time	141,913 (59.13)	136,296 (59.41)	2811 (55.62)	2806 (58.37)	
1–2 times	64,760 (27.06)	62,011 (27.03)	1511 (29.90)	1238 (25.75)	
3–4 times	21,364 (8.93)	20,418 (8.90)	491 (9.72)	455 (9.47)	
≥ 5 times	11,250 (4.70)	10,701 (4.66)	241 (4.77)	308 (6.41)	
Exercise, days per week					0.398
< 1 day	59,871 (25.02)	57,433 (25.03)	1149 (22.73)	1289 (26.82)	
1–4 days	108,348 (45.28)	103,860 (45.27)	2353 (46.56)	2135 (44.41)	
≥ 5 days	71,068 (29.70)	68,133 (29.70)	1552 (30.71)	1383 (28.77)	
Body mass index					< 0.001
Underweight (< 18.5 kg/m^2^)	5548 (2.32)	5280 (2.30)	129 (2.55)	139 (2.89)	
Normal range (18.5–23 kg/m^2^)	90,072 (37.64)	86,733 (37.80)	1782 (35.26)	1577 (32.39)	
Overweight (23–25 kg/m^2^)	67,809 (28.34)	65,153 (28.40)	1435 (28.39)	1221 (25.40)	
Obese (≥ 25 kg/m^2^)	75,858 (31.70)	72,260 (31.50)	1708 (33.80)	1890 (39.32)	
Hypertension	128,316 (53.62)	122,251 (53.29)	2906 (57.50)	3159 (65.72)	< 0.001
Systolic blood pressure, mmHg	124.27 ± 15.12	124.17 ± 15.04	125.25 ± 15.89	128.34 ± 17.41	< 0.001
Family history of diabetes, yes	17,836 (7.45)	17,060 (7.44)	434 (8.59)	342 (7.11)	0.539
Laboratory findings					
Fasting glucose, mg/dL	94.13 ± 11.20	94.07 ± 11.17	95.05 ± 11.48	95.91 ± 11.91	< 0.001
Estimated glomerular filtration rate, mL/min/1.73 m^2^	83.66 ± 34.40	83.87 ± 34.43	79.60 ± 36.53	77.92 ± 29.18	< 0.001

Data are represented as the number of participants (%) or mean ± standard deviation. ^†^*P* for trends are derived from the Mantel–Haenszel test for linear trend and the Jonckheere’s trend test

### Risk of T2DM according to proteinuria

**Fig. 2:**
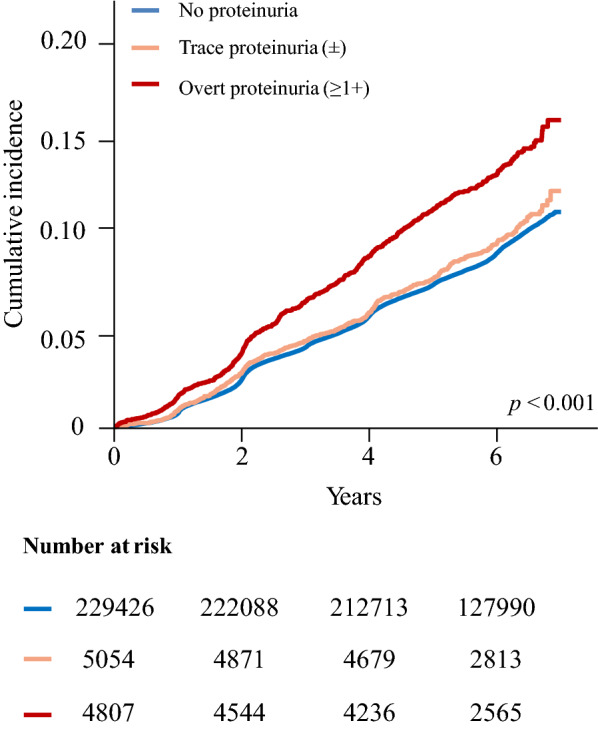
Kaplan Meier curves for the Event Free Survival for Development of Type 2 Diabetes Mellitus according to Dipstick Proteinuria. *P* value is derived by a log-rank test

During the mean follow-up period of 5.73 (standard deviation, 1.18) years, 22,215 participants had new-onset T2DM. A Kaplan–Meier curve for the cumulative risk of new-onset T2DM showed an increased risk for T2DM according to the presence and degree of proteinuria (Fig. 2). In the univariate Cox regression models, the risk of new-onset T2DM was significantly higher in the overt proteinuria group than in the group without proteinuria (crude HR: 1.50; 95% CI: 1.38, 1.62). This positive association with proteinuria was maintained in the fully adjusted model (adjusted HR: 1.19; 95% CI: 1.10, 1.29) (Table 2).

**Table 2:** Risk of new-onset type 2 diabetes mellitus according to degree of proteinuria

Degree of proteinuria	Case^a^	HR (95% CI)			
		Crude model	Model 1	Model 2	Model 3
No proteinuria	21,086	1 (Ref)	1 (Ref)	1 (Ref)	1 (Ref)
Trace proteinuria (±)	491	1.06 [0.97–1.16]	1.04 [0.95–1.14]	1.01 [0.92–1.10]	0.96 [0.88–1.05]
Overt proteinuria (≥ 1 +)	638	1.50 [1.38–1.62]	1.46 [1.35–1.58]	1.32 [1.22–1.42]	1.19 [1.10–1.29]

Model 1: adjusted for sex, and age

Model 2: adjusted for sex, age, current smoking, physical activity, alcohol consumption, family history of diabetes, body mass index, and systolic blood pressure

Model 3: adjusted for sex, age, current smoking, physical activity, alcohol consumption, family history of diabetes, body mass index, systolic blood pressure, fasting glucose level, and estimated glomerular filtration rate

*HR* hazard ratio, *CI* indicates confidence interval

^a^New-onset type 2 diabetes mellitus

### Risk of T2DM according to proteinuria and fasting glucose level

To investigate whether the association between proteinuria and new-onset T2DM risk differed according to risk factors, subgroup analyses were performed (Additional file 1: Fig. S1). In sex, age, and other subgroups, significant interaction effects with proteinuria for T2DM risk were nor found (*P* > 0.05). To further evaluate whether there were interaction effects between proteinuria and baseline fasting glucose levels, we classified participants according to their baseline fasting glucose levels into the normal fasting glucose group (< 100 mg/dL) and the impaired fasting glucose group (≥ 100 mg/dL and < 126 mg/dL). In both groups, overt proteinuria was significantly associated with increased risk for new-onset T2DM compared with no proteinuria (Additional file 2: Table S1).

### Risk of T2DM according to the change in proteinuria status

We further analyzed the risk for new-onset T2DM according to the change in proteinuria status. For this analysis, we collected data on participants who also underwent a health examination between 2002 and 2003 that included a dipstick proteinuria test (n = 238,679). The participants were divided into four groups according to the change in proteinuria: Negative/Trace → Negative/Trace (− → −; Group 1), Negative/Trace →  ≥ 1 + (− →  + ; Group 2), ≥ 1 +  → Negative/Trace (+ → −; Group 3), ≥ 1 +  →  ≥ 1 + (+ →  + ; Group 4). In the analyses, group 1 (− → −) had lower risk for new-onset T2DM compared with the other three groups (Table 3). Change in lifestyle characteristics (smoking status, physical activity, alcohol consumption) of study participants according to change in proteinuria status are shown in Additional file 3: Table S2.

**Table 3:** Risk of type 2 diabetes mellitus risk according to change in proteinuria status

Change in proteinuria	Participants (number)	Case^a^	HR (95% CI)			
			Crude model	Model 1	Model 2	Model 3
Negative/Trace → Negative/Trace	231,223	21,249	1 (Ref)	1 (Ref)	1 (Ref)	1 (Ref)
Negative/Trace → ≥ 1 +	4380	569	1.46 [1.35–1.59]	1.44 [1.32–1.56]	1.30 [1.19–1.41]	1.18 [1.08–1.28]
≥ 1 + → Negative/Trace	2660	276	1.15 [1.02–1.29]	1.19 [1.05–1.33]	1.12 [0.99–1.26]	1.13 [1.01–1.28]
≥ 1 + → ≥ 1 +	416	68	1.88 [1.48–2.39]	1.77 [1.40–2.25]	1.53 [1.20–1.94]	1.39 [1.10–1.76]

Model 1: adjusted for sex and age

Model 2: adjusted for sex, age, current smoking, physical activity, alcohol consumption, family history of diabetes, body mass index, systolic blood pressure

Model 3: adjusted for sex, age, current smoking, physical activity, alcohol consumption, family history of diabetes, body mass index, systolic blood pressure, fasting glucose level, and estimated glomerular filtration rate

*HR* hazard ratio, * CI *indicates confidence interval

^a^New-onset type 2 diabetes mellitus

## Discussion

This cohort study investigated the association between dipstick proteinuria and the risk of new-onset T2DM using the nationwide population-based health screening database in Korea. Participants with proteinuria had a higher risk of developing T2DM independent of other risk factors, including fasting glucose level. There was a dose-dependent increase in the risk of T2DM according to the degree of dipstick proteinuria. The urine dipstick test is already a widely used screening tool for proteinuria and other urinary issues [24]. Our study implies that the dipstick proteinuria test may also be useful for identifying patients at high risk of developing T2DM.

There are several potential mechanisms that may help explain the relationship between proteinuria and increased T2DM risk. The first is that proteinuria is a sensitive marker of inflammation, and sustained proteinuria is considered a cause of further inflammation and oxidative stress [25, 26]. Numerous studies have documented a strong positive relationship between proteinuria and inflammatory biomarkers such as C-reactive protein, TNF-α, interleukin-6, and monocyte chemotactic protein-1 [27, 28]. In a large cohort of chronic kidney disease patients, the inflammation score was positively associated with proteinuria, regardless of estimated glomerular filtration rate [29]. Recent evidence has suggested that proteinuria itself and underlying medical conditions can elicit pro-inflammatory effects and the secretion of inflammatory cytokines including monocyte chemotactic protein-1, endothelin-1, and nuclear factor-_k_B [30, 31]. Secretion of these chemokines and inflammatory cytokines has been shown to induce insulin resistance, a major hallmark of T2DM pathogenesis [32–34]. That is to say, elevated levels of the inflammatory biomarkers are predictors of the development of T2DM [35, 36]. The implications of systemic inflammation and proteinuria may explain the high risk of T2DM in patients with proteinuria.

Microvascular endothelial dysfunction is a typical clinical feature found in patients with proteinuria [36, 37]. β-cells of the pancreatic islets secrete insulin and play a major role in glycemic control and the development of diabetes [38]. The pancreatic islet is highly vassalized with an extensive capillary network, which is essential for the survival and function of islet β-cells [39]. The islet endothelial dysfunction contributes to impaired β-cell function in diabetes [40]. Furthermore, impaired endothelial permeability disrupts insulin delivery to the skeletal muscle, liver, or adipose tissue, thereby limiting insulin action [41]. Many epidemiological studies have reported that biomarkers of endothelial dysfunction could be predictors of T2DM development [9, 42]. Although it is uncertain whether endothelial dysfunction is a consequence or preceding factor of T2DM [12], the link between endothelial dysfunction and diabetes may explain the increased risk of T2DM in patients with proteinuria, the result of current study.

The kidneys play a central role in glucose homeostasis, and impaired renal proximal tubules may be another contributing factor in the development of T2DM in patients with proteinuria [43]. The kidneys filter approximately 180 g of glucose per day, and glucose reabsorption in the kidney is primarily mediated by sodium glucose cotransporters 1 and 2 in the proximal tubule, which help regulate blood glucose levels [44]. The proximal tubule is also a major site of albumin reabsorption in the kidneys [45]. Damaged proximal tubules, leading to the presence of proteinuria, may interfere with the reabsorption of glucose and regulation of glucose homeostasis.

Due to the limitation of the observational design without interventions, we could not conclude whether proteinuria had a causal effect on the risk of T2DM (mediator) or that proteinuria only reflected an underlying inflammatory or other deteriorating condition associated with an increased risk of T2DM (risk marker). However, substantial clinical and experimental evidence has suggested that systemic inflammation and endothelial dysfunction can contribute to the pathogenesis and development of T2DM, suggesting they are risk factors for diabetes [10, 46]. Therefore, we supposed that proteinuria may be a potential treatment target for reducing the development of T2DM and improving glycemic control. The findings of the current study, namely, the dose–response increase in T2DM risk according to the degree of proteinuria and the concurrent change in the risk according to the change in proteinuria, suggest that proteinuria reduction would have a beneficial effect on the prevention of diabetes. Intense exercise, heat waves and extreme temperatures, and consumption of dietary protein in excess of recommended amounts accelerate a progressive loss of renal capacity leading to proteinuria [47–49]. However, we could not consider these variable due to unavailability of such information from the health-insurance database. In addition, modifications in lifestyle factors, anti-proteinuric therapies and anti-hypertensive therapies such as angiotensin-converting enzyme inhibitors or angiotensin receptor blockers have been reported to have an additive effect on the reduction of proteinuria [50–52]. Thus, further research is needed to evaluate the effect of these interventions on reducing proteinuria to aid in the prevention of T2DM.

We should acknowledge several limitations of current study. First, current study had a retrospective observational design without interventions; it was therefore difficult to explain the exact mechanism between proteinuria and the risk for new-onset T2DM. Second, urine dipstick proteinuria was assessed by a single measurement, which may have a measurement error. Third, as our study’s participants were limited to the Korean population, the results should be interpreted with caution. Nonetheless, this study was strengthened by its use of large-scale reliable data from the NHIS, representing nationwide health information on the Korean population, and its evaluation of the long-term risk for new-onset T2DM through both health claims data and serially performed health screening examinations.

## Conclusions

In conclusion, the present study found that dipstick proteinuria was an independent risk factor for the development of new-onset T2DM. The risk of T2DM increased proportionally with the severity of dipstick proteinuria. Further large-scale clinical and experimental studies should be conducted to investigate the exact mechanisms between of them and to determine whether proteinuria is a potential treatment target for the prevention and control of T2DM.

## Supplementary Information

**Additional file 1: Fig. S1.** Effect of proteinuria on type 2 diabetes mellitus in each subgroup by risk factor.

**Additional file 2: Table S1.** Effect of proteinuria on type 2 diabetes mellitus according to the baseline fasting glucose level.

**Additional file 3: Table S2.** Change in lifestyle characteristics of study participants according to change in proteinuria status.

## Data Availability

The dataset (NHIS-HEALS) supporting the conclusion of this study is available from the National Health Insurance Sharing Service (http://nhiss.nhis.or.kr/bd/ab/bdaba021eng.do). To gain access to the dataset, a completed application form, research proposal, and an applicant’s approval document from the institutional review board should be submitted to and reviewed by the inquiry committee of research support in National Health Insurance Sharing Service.

## Abbreviations

T2DM: Type 2 diabetes mellitus
NHIS-HEALS: National Health Insurance Service-Health Screening Cohort
HR: Hazard ratio
CI: Confidence interval
