# Supplementary material for: Dipstick proteinuria and risk of type 2 diabetes mellitus: a nationwide population-based cohort study
Source: J Transl Med. 2021 Jun 26;19:271. doi: 10.1186/s12967-021-02934-y (PMC8235563; doi:10.1186/s12967-021-02934-y)
Supplement: Supplementary file 1 — Additional file 1: Fig. S1. Effect of proteinuria on type 2 diabetes mellitus in each subgroup by risk factor. [file 12967_2021_2934_MOESM1_ESM.pdf]

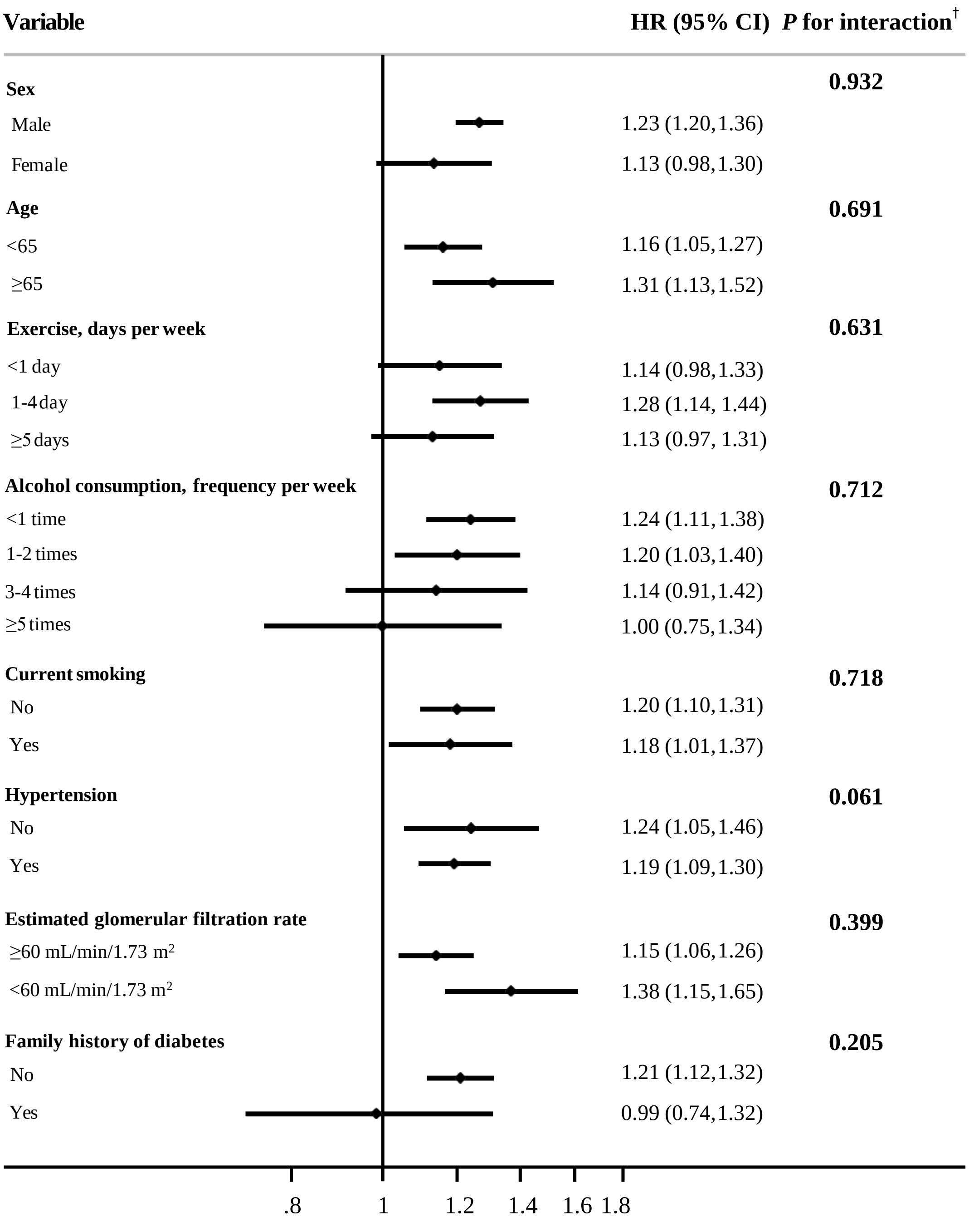

Fig. S1 Effect of proteinuria on type 2 diabetes mellitus in each subgroup by risk factor. Data show hazard ratio (HR) and 95% confidence intervals (CI) for proteinuria derived from Cox proportional hazard regression model for the development of type 2 diabetes mellitus. †*P* for interaction between risk factors and proteinuria on the risk of type 2 diabetes.
