# Supplementary material for: Dipstick proteinuria and risk of type 2 diabetes mellitus: a nationwide population-based cohort study
Source: J Transl Med. 2021 Jun 26;19:271. doi: 10.1186/s12967-021-02934-y (PMC8235563; doi:10.1186/s12967-021-02934-y)
Supplement: Supplementary file 2 — Additional file 2: Table S1. Effect of proteinuria on type 2 diabetes mellitus according to the baseline fasting glucose level. [file 12967_2021_2934_MOESM2_ESM.pdf]

Table S1 Effect of proteinuria on type 2 diabetes mellitus according to the baseline fasting glucose level

Degree of proteinuria	Fasting glucose level	
	**< 100 mg/dL**	**≥ 100 mg/dL and < 126 mg/dL**
No proteinuria	1 (Ref)	1 (Ref)
Trace proteinuria (±)	0.87 [0.74-1.01]	1.03 [0.92-1.15]
Overt proteinuria (≥1+)	1.45 [1.28-1.64]	1.13 [1.02-1.25]

Data are adjusted hazard ratio [95% confidence interval] derived from multivariate Cox proportional hazard regression model. Models are adjusted for sex, age, current smoking, physical activity, alcohol consumption, family history of diabetes, body mass index, systolic blood pressure, and estimated glomerular filtration rate.
