# Supplementary material for: Dipstick proteinuria and risk of type 2 diabetes mellitus: a nationwide population-based cohort study
Source: J Transl Med. 2021 Jun 26;19:271. doi: 10.1186/s12967-021-02934-y (PMC8235563; doi:10.1186/s12967-021-02934-y)
Supplement: Supplementary file 3 — Additional file 3: Table S2. Change in lifestyle characteristics of study participants according to change in proteinuria status. [file 12967_2021_2934_MOESM3_ESM.pdf]

Table S2 Change in lifestyle characteristics of study participants according to change in proteinuria status

**Change in lifestyle**	**Change in proteinuria**			***P* for trend**†
	Increase (Negative/Trace → ≥1+) N=4380	No change (Negative/Trace → Negative/Trace or ≥1+ → ≥1+) N=231639	Decrease (≥1+ → Negative/Trace) N=2660	
Smoking status				0.104
Stop smoking	387 (8.84)	19136 (8.26)	187 (7.03)	
No change	3831 (87.47)	203258 (87.75)	2390 (89.85)	
Start smoking	162 (3.70)	9245 (3.99)	83 (3.12)	
Alcohol consumption				0.003
Decrease	730 (16.67)	39127 (16.89)	474 (17.82)	
No change	2929 (66.87)	156898 (67.73)	1836 (69.02)	
Increase	721 (16.46)	35614 (15.37)	350 (13.16)	
Physical activity				0.204
Decrease	476 (10.87)	23392 (10.10)	287 (10.79)	
No change	1779 (40.62)	92375 (39.88)	1052 (39.55)	
Increase	2125 (48.52)	115872 (50.02)	1321 (49.66)	

Data are represented as the number of participants (%).

†*P* for trends are derived from the Mantel-Haenszel test for linear trend.
